# Efficacy and safety of combination antifungal therapy in Korean haematological patients with invasive aspergillosis

**DOI:** 10.1111/myc.12972

**Published:** 2019-08-18

**Authors:** Dong‐Gun Lee, Hye‐Jung Lee, Jean Li Yan, Stephen Sheng‐Fong Lin, Jalal A. Aram

**Affiliations:** ^1^ Division of Infectious Diseases Department of Internal Medicine College of Medicine The Catholic University of Korea Seoul South Korea; ^2^ Pfizer Inc Seoul South Korea; ^3^ Pfizer Inc New York NY USA

**Keywords:** anidulafungin, combination antifungals, echinocandins, galactomannan, haematologic malignancies, immunocompromised, invasive aspergillosis, voriconazole

## Abstract

This randomised, double‐blind, placebo‐controlled trial assessed the efficacy, safety and tolerability of voriconazole+anidulafungin (combination) or voriconazole+placebo (monotherapy) for invasive aspergillosis (IA; NCT00531479). We present a post hoc analysis of Korean and non‐Korean patients with IA (including baseline positive serum galactomannan [GM]). Immunocompromised patients ≥ 16 years with IA were randomised 1:1, combination or monotherapy, for ≥ 2 weeks’ treatment. The primary endpoint was 6‐ and 12‐week all‐cause mortality (Korean modified intent‐to‐treat [mITT] population). Overall, 454 patients enrolled (Koreans: 56 [combination: 28, monotherapy: 28], non‐Koreans: 398 [combination: 200, monotherapy: 198]). The mITT population comprised 40 Koreans (combination: 23; monotherapy: 17) and 237 non‐Koreans (combination: 112; monotherapy: 125). Week 6 treatment difference in mortality rate between combination and monotherapy was −6.4% in non‐Koreans. This reduction was more marked in Koreans (−22.4%). Week 12 difference in all‐cause mortality between combination and monotherapy was −17.7% (Koreans) and −20.2% at Week 6 (Koreans; positive baseline GM). Week 6 mortality (Koreans [mITT]; baseline GM >0.5‐2.0) was 0/13 (combination) and 2/6 (monotherapy). Serious adverse events were numerically higher for combination than monotherapy (Koreans: 57.1%, 46.4%; non‐Koreans: 49.5%, 46.0%). In Koreans, combination therapy was associated with marginally better outcomes than monotherapy and more so than in non‐Koreans.

## 1 INTRODUCTION

Invasive aspergillosis (IA) is an important cause of morbidity and mortality in patients with haematologic malignancies, as well as those who have undergone allogeneic haematopoietic stem cell transplantation (HSCT).^1^, ^2^, ^3^ Risk factors for mortality in patients with IA following HSCT include the degree of compromised immunity and dissemination of infection, thus suggesting the importance of prompt diagnosis, initiation of therapy and reduction in immunosuppression after diagnosis.^3^

Treatment with amphotericin B deoxycholate has been shown to be effective in the treatment of patients with IA but its use may be limited by nephrotoxicity.^4^ In a prospective, randomised study, voriconazole led to better outcomes compared with amphotericin B deoxycholate as primary therapy for IA at Week 12 in the modified intent‐to‐treat (mITT) population^5^ and is now the initial standard treatment for IA.^6^

In vitro studies have demonstrated synergistic activity of voriconazole and echinocandins against *Aspergillus* species.^7^, ^8^, ^9^, ^10^ In patients with IA who experienced failure of initial therapy with amphotericin B formulations, a combination of voriconazole and caspofungin has been shown to improve survival compared with voriconazole alone, with fewer patients dying of IA 3 months after receipt of salvage therapy.^11^ However, other studies have demonstrated no survival advantage among patients who received combination therapy with voriconazole and caspofungin compared with voriconazole monotherapy.^3^, ^12^

More recently, a randomised, double‐blind, placebo‐controlled trial assessed the safety and efficacy of voriconazole and the echinocandin anidulafungin, compared with voriconazole monotherapy in the treatment of IA.^13^ Treatment of IA with the combination therapy was associated with a clinically meaningful survival benefit vs voriconazole monotherapy in patients with haematologic malignancies or HSCT. Most patients in the investigation (78.7%) had a diagnosis of IA established by radiographic findings and maximum galactomannan (GM) antigen positivity. GM measurement is a non‐invasive tool for IA diagnosis among at‐risk patients and is included as one of the mycological criteria for a probable case of IA case definition by the consensus group of the European Organisation for Research and Treatment of Cancer/Mycosis Study Group (EORTC/MSG).^14^ Serial GM measurements have been investigated for monitoring treatment response or as a surrogate endpoint to assess clinical outcomes in patients with IA.^15^, ^16^, ^17^

In the present study, we report the results of a post hoc analysis of data from the Korean sub‐population of a primary double‐blind, randomised study.^13^ The rationale for evaluating this sub‐group is that about 13%‐23% of the Asian population inherit a cytochrome P450 2C19 (CYP2C19) poor metabolizer genotype compared with 3%‐5% observed in Caucasians.^18^, ^19^, ^20^, ^21^, ^22^ As voriconazole is mainly metabolised by CYP2C19, which contains known polymorphisms (eg poor metabolizer genotype) that reduce the activity of this enzyme, data on the response to treatment in this sub‐population were explored.^23^, ^24^, ^25^

Therefore, this post hoc analysis aimed to evaluate the efficacy and safety of voriconazole and anidulafungin combination therapy and voriconazole monotherapy in Korean and non‐Korean patients with IA, including those with a positive serum GM at baseline.

## 2 PATIENTS AND METHODS

The present investigation was a post hoc analysis of sub‐groups of Korean and non‐Korean patients who were included in a randomised controlled trial of the effectiveness and safety of a combination of anidulafungin and voriconazole, and voriconazole alone, for the treatment of IA. The authors confirm that the ethical policies of the journal, as noted on the journal's author guidelines page, have been adhered to and the appropriate ethical review committee approval has been received. The study was conducted in accordance with the International Conference on Harmonization Good Clinical Practice Guidelines and ethical principles originating in or derived from the Declaration of Helsinki, approved by the appropriate institutional review boards, and registered on ClinicalTrials.gov (primary study: NCT00531479). Written informed consent was obtained for all patients.

### 2.1 Patient eligibility (primary study)

Briefly, patients were included if they were male or female, aged ≥ 16 years and immunocompromised because of allogeneic HSCT after myeloablative/non‐myeloablative conditioning or haematologic malignancies.^13^ Patients had to be diagnosed with proven, probable or possible IA as revised by the modified EORTC/MSG consensus definitions^14^; (Appendix S1: Table S1). Any patients enrolled with possible IA had to have a proven or probable diagnosis established within 7 days of enrolment.

Among the reasons for excluding patients were progressive haematologic disease that was unlikely to respond to treatment; systemic anti‐mould antifungals for treatment of IA for ≥ 4 days; severe liver dysfunction (>5× upper limit of normal); or a Karnofsky score <20. Other criteria included anticipated death within 30 days due to non‐infectious factors, those who required mechanical ventilation, were pregnant or lactating, had received interacting drugs (eg rifampin), or were allergic or experienced serious reactions to azoles or echinocandins.^13^

### 2.2 Design and endpoints (primary study)

The primary study was a randomised, double‐blind, placebo‐controlled multicentre trial in which patients were randomised 1:1 to either voriconazole plus anidulafungin (combination therapy) or voriconazole plus placebo (monotherapy).^13^ The primary endpoint was all‐cause mortality at 6 weeks in patients with proven or probable IA in the mITT group. The mITT population included all randomised patients with proven or probable IA confirmed by Day 7 following enrolment, adjudicated by a data review committee (DRC), and who received at least one dose of study drug. Treatment randomisation (Appendix S1: Figure S1) and secondary endpoints are described in Appendix S1.^13^

### 2.3 Patients (post hoc analysis)

In the present analysis, a sub‐group of patients from the primary study were included, with the objective of comparing the efficacy and safety of combination therapy or monotherapy in Korean and non‐Korean patients. Patients were included if they had been randomised to receive voriconazole and anidulafungin in combination or voriconazole monotherapy (plus placebo) for a minimum of 2 weeks and met the criteria for the mITT population. Participants had to have a proven or probable IA diagnosis, based on EORTC/MSG criteria and confirmed by an independent DRC, and must have received therapy.

### 2.4 Endpoints (post hoc analysis)

The primary endpoint was all‐cause mortality at Week 6 in the Korean mITT population. In addition, sub‐group analyses of all‐cause mortality at Weeks 6 and 12 were performed in Korean and non‐Korean mITT patients.

A GM screening assay has been evaluated as a potential diagnostic tool for early diagnosis of IA in patients receiving allogeneic HSCT.^15^ In this analysis, we also examined all‐cause mortality at Week 6 in Korean and non‐Korean mITT patients who had a positive serum GM or bronchoalveolar lavage (BAL) GM at baseline.

The safety and tolerability of combination therapy and monotherapy were assessed in Korean and non‐Korean patients in terms of the number of patients with treatment‐emergent adverse events (TEAEs) of all causalities by system organ class and Medical Dictionary for Regulatory Activities (MedDRA)‐preferred terms. In addition, all‐cause mortality was evaluated in the safety population at Weeks 6 and 12 in the Korean and non‐Korean sub‐groups.

### 2.5 Statistical methods

Statistical methods for the primary analysis, including a review of safety data, have been reported elsewhere.^13^

Mortality rate was based on the Kaplan‐Meier (KM) product limit estimator. Treatment difference (stratified) was based on a weighted difference in proportions (*d*), using the following formula: $d=\frac{\sum_{i}w_{i}\left(P_{\mathrm{Ci}}-P_{\mathrm{Vi}}\right)}{\sum_{i}w_{i}}$, where *P*_*Ci*_ and *P*_*Vi*_ represented the KM rates for combination and monotherapy, w_*i*_ was the harmonic mean of the sample sizes for stratum *i,* calculated by: $w_{i}=\frac{n_{\mathrm{Ci}}n_{\mathrm{Vi}}}{\left(n_{\mathrm{Ci}}+n_{\mathrm{Vi}}\right)}$, where *n*_*C*_ and *n*_*V*_ were the number of patients in the combination and monotherapy groups, respectively. Confidence intervals (95% CI) were calculated using Greenwood's formula for the variance of the KM estimator. *P*‐values were based on a one‐sided test.

The safety and tolerability of combination therapy and monotherapy in Korean and non‐Korean sub‐groups were summarised by descriptive statistics, including overall numbers of adverse events (AEs), patients with AEs, TEAEs, discontinuations due to AEs or requiring dose reduction.

## 3 RESULTS

### 3.1 Patient characteristics

Out of 454 patients enrolled, 56 were Korean and 398 were non‐Korean. Patient baseline characteristics in both populations are shown in Table 1. The safety population of Korean patients comprised 28 who received combination therapy and 28 who received monotherapy. In the non‐Korean group, 200 and 198 patients received combination and monotherapy, respectively.

**Table 1:** Patient characteristics of Korean and non‐Korean populations at baseline

	Voriconazole + anidulafungin			Voriconazole + placebo		
	Male	Female	Total	Male	Female	Total
**Korean patients, N**	**16**	**12**	**28**	**19**	**9**	**28**
Age, years (n)						
<18	0	0	0	0	0	0
18‐44	8	5	13	6	2	8
45‐64	6	6	12	10	6	16
≥65	2	1	3	3	1	4
Mean (SD)	44.9 (16.1)	50.2 (12.0)	47.2 (14.5)	49.8 (14.6)	50.8 (11.8)	50.1 (13.5)
Range	19‐73	28‐69	19‐73	22‐70	27‐66	22‐70
Weight, kg						
Mean (SD)	64.4 (18.8)	53.0 (9.0)	59.5 (16.2)	64.0 (7.1)	52.4 (7.5)	60.3 (9.0)
Range	36.9‐101.8	39.0‐67.5	36.9‐101.8	54.0‐76.9	39.5‐63.0	39.5‐76.9
Body mass index, kg/m^2^						
Mean (SD)	21.8 (5.1)	22.2 (3.2)	22.0 (4.3)	22.8 (2.7)	21.9 (2.6)	22.5 (2.7)
Range	12.9‐31.6	18.5‐29.1	12.9‐31.6	18.8‐27.6	16.7‐25.3	16.7‐27.6
Height, cm						
Mean (SD)	170.8 (7.6)	154.5 (6.9)	163.8 (10.9)	167.8 (6.4)	154.5 (4.6)	163.6 (8.6)
Range	161.0‐182.5	143.1‐164.4	143.1‐182.5	157.5‐183.3	147.7‐160.8	147.7‐183.3
**Non‐Korean patients, N**	**118**	**82**	**200**	**112**	**86**	**198**
Age, years						
<18	0	0	0	0	1	1
18‐44	29	23	52	28	32	60
45‐64	60	41	101	48	42	90
≥65	29	18	47	36	11	47
Mean (SD)	52.4 (16.0)	53.0 (14.8)	52.7 (15.5)	53.6 (15.9)	47.9 (16.1)	51.1 (16.2)
Range	18‐83	18‐79	18‐83	18‐83	17‐73	17‐83
Race, n						
White	98	69	167	90	67	157
Black	3	2	5	2	1	3
Asian	13	9	22	16	13	29
Other	4	2	6	4	5	9
Weight, kg						
Mean (SD)	75.5 (15.6)	64.6 (16.5)	71.0 (16.8)	76.3 (16.2)	63.2 (14.8)	70.6 (16.9)
Range	42.0‐116.8	35.0‐111.3	35.0‐116.8	45.0‐150.0	39.5‐112.0	39.5‐150.0
Body mass index, kg/m^2^						
Mean (SD)	24.3 (4.6)	24.8 (5.6)	24.5 (5.1)	24.7 (4.5)	24.2 (5.5)	24.5 (4.9)
Range	15.1‐39.0	15.0‐41.0	15.0‐41.0	16.6‐47.3	14.9‐44.9	14.9‐47.3
N	115	82	197	110	86	196
Height, cm						
Mean (SD)	175.7 (8.0)	161.0 (7.4)	169.6 (10.6)	174.9 (8.5)	161.5 (7.0)	169.1 (10.3)
Range	155.0‐192.0	139.0‐175.3	139.0‐192.0	155.0‐201.0	145.0‐178.0	145.0‐201.0
N	115	82	197	110	86	196

Abbreviation: SD, standard deviation.

The mITT population included 40 Korean (combination therapy n = 23; monotherapy n = 17) and 237 non‐Korean patients (combination therapy n = 112; monotherapy n = 125). Overall, Korean and non‐Korean patients had comparable baseline characteristics except for a numerically higher height, weight and body mass index in the non‐Korean sub‐group (Table 1). Among Korean patients, the baseline demographic characteristics were comparable in the two treatment arms.

### 3.2 Efficacy endpoints

All‐cause mortality in the mITT for Korean patients at Week 6 was 17.6% (4/23) in the combination therapy arm and 41.2% (7/17) in the monotherapy arm (treatment difference [stratified, based on a weighted difference in proportions]): −22.4%; 95% CI, −52.3 to 7.6%). At Week 12, all‐cause mortality in the same sub‐group was 26.8% (6/23) in the combination therapy arm and 47.1% (8/17) in the monotherapy arm (treatment difference [stratified]: −17.7%; 95% CI, −49.0 to 13.6%).

In the sub‐group analysis of non‐Korean patients (mITT), mortality rate at Week 6 was 19.8% (22/112) in the combination arm and 26.0% (32/125) in the monotherapy arm (difference: −6.4%; 95% CI, −17.4 to 4.6%). This reduction was more marked in Koreans (−22.4%). At Week 12, mortality was 29.9% (33/112) in the combination arm and 38.3% (47/125) in the monotherapy arm in non‐Korean patients (treatment difference [stratified]: −8.4%; 95% CI, −20.6 to 3.8%). The difference in all‐cause mortality at Week 12 between combination therapy and monotherapy was also greater in Koreans than in non‐Koreans (−17.7% and −8.4%; Table 2).

**Table 2:** All‐cause mortality at Weeks 6 and 12 in Korean and non‐Korean patients (mITT population)

	All‐cause mortality (week 6)		All‐cause mortality (week 12)	
	Korean patients	Non‐Korean patients	Korean patients	Non‐Korean patients
Voriconazole + anidulafungin	17.6% (4/23)	19.8% (22/112)	26.8% (6/23)	29.9% (33/112)
Voriconazole + placebo	41.2% (7/17)	26.0% (32/125)	47.1% (8/17)	38.3% (47/125)
Difference^a^	−22.4%	−6.4%	−17.7%	−8.4%
95% CI	−52.3% to 7.6%	−17.4 to 4.6%	−49.0 to 13.6%	−20.6 to 3.8%

Abbreviations: CI, confidence interval; mITT, modified intent‐to‐treat.

^a^Treatment difference (stratified) is based on a weighted difference in proportions.

**Figure 1:**
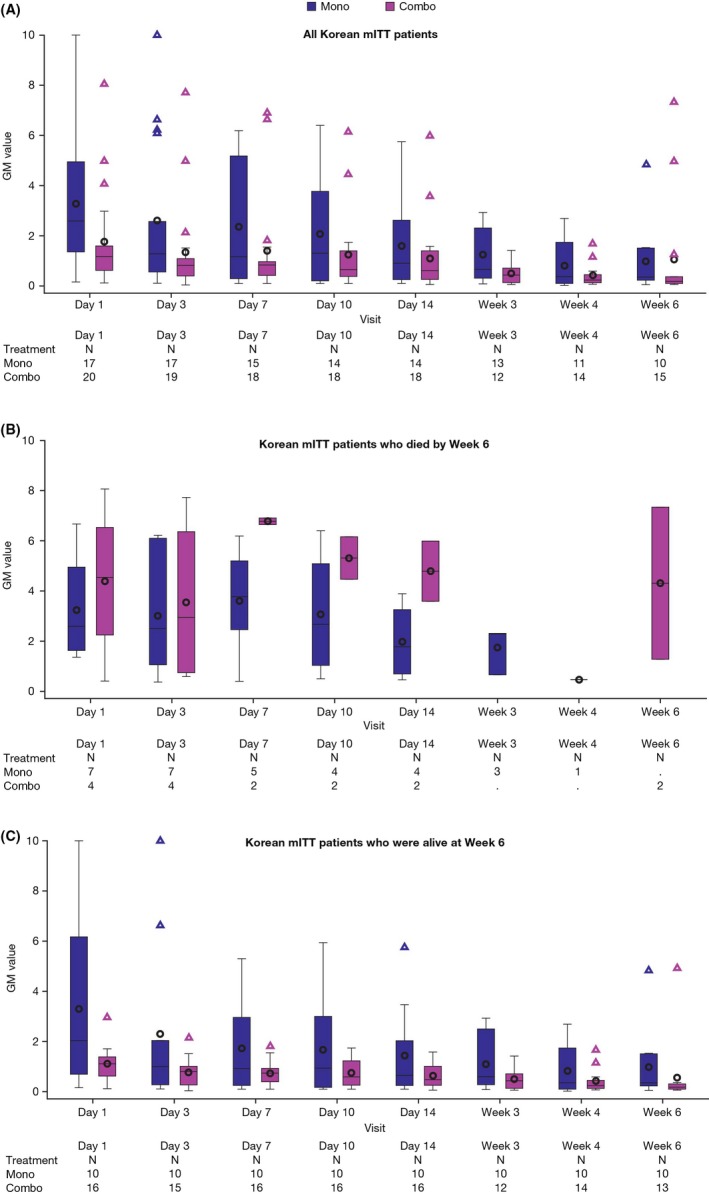
Serum GM and mortality in Korean patients in combination and monotherapy arms. Maximum serum GM values by combination and monotherapy in (A) all Korean mITT patients, (B) Korean mITT patients who died by Week 6 and (C) Korean mITT patients who were alive at Week 6. Includes patients with non‐zero values at baseline. Horizontal line, median; o, mean; box, Q1‐Q3; whiskers, 5‐95 percentile; Δ, outliers (outside 5‐95 percentile). combo, voriconazole + anidulafungin combination therapy; GM, galactomannan; mITT, modified intent‐to‐treat; mono, voriconazole + placebo monotherapy

The majority of patients in the Korean sub‐group had serum or BAL GM‐positive baseline tests (combination therapy: 71.4% [20/28]; monotherapy: 60.7% [17/28]). All‐cause mortality at Week 6 in Korean patients (mITT) who had a positive serum GM or BAL GM at baseline was 20.3% (4/20) after combination therapy and 41.2% (7/17) after monotherapy (treatment difference [stratified]: −20.2%; 95% CI, −50.8 to 10.5%; Table 3). For baseline GM values in the range of > 0.5‐2.0, mortality at Week 6 in Korean patients (mITT) was 0/13 after combination therapy and 2/6 after monotherapy. For baseline GM values ≤ 0.5, mortality at Week 6 was 1/3 after combination therapy and 0/1 after monotherapy, and for baseline GM values > 2.0, it was 3/4 after combination therapy and 5/10 after monotherapy. Maximum serum GM values in the Korean mITT population between Day 1 and Week 6 were generally higher in the monotherapy arm than the combination therapy arm (Figure 1A). GM values were also generally higher in patients who died by Week 6 vs those who were alive at Week 6 in the combination arm (Figure 1B,C). In the overall Korean mITT population, GM values showed a general decline over time compared with baseline in the combination therapy and monotherapy arms regardless of survival outcomes (Figure 1A,B). The non‐Korean sub‐group with positive serum or BAL GM at baseline had all‐cause mortality rates at Week 6 of 15.0% and 24.9% in the two treatment arms, respectively (treatment difference [stratified]: −9.94%; 95% CI, −21.6 to 1.7%; Table 3).

**Table 3:** Serum galactomannan (GM) or bronchoalveolar lavage GM levels and all‐cause mortality at 6 weeks in Korean and non‐Korean patients with positive serum GM or BAL GM at baseline (mITT)

	Voriconazole + anidulafungin		Voriconazole + placebo		Between‐group differences,^a^ % (95% CI)^b^	
	Korean patients	Non‐Korean patients	Korean patients	Non‐Korean patients	Korean patients	Non‐Korean patients
Serum and BAL GM						
Evaluated for serum only, n	17	44	15	49	N/A	N/A
Evaluated for BAL only, n	3	37	2	32		
Evaluated for serum and BAL, n	0	7	0	12		
GM < 0.5, n	3	39	2	46	N/A	N/A
GM 0.5‐2.0, n	13	33	5	33		
GM > 2.0, n	4	11	10	12		
Patients, n	20	88	17	93	−20.2 (−50.8 to 10.5)	−9.94 (−21.6 to 1.7)
Mortality rate,^c^ %	20.3	15.0	41.2	24.9		

Abbreviations: BAL, bronchoalveolar lavage; CI, confidence interval; GM, galactomannan; KM, Kaplan‐Meier; mITT, modified intent‐to‐treat; N/A, not applicable.

^a^Treatment difference (stratified) is based on a weighted difference in proportions.

^b^95% CI is calculated using Greenwood's formula for the variance of the KM estimator.

^c^Mortality rate is based on the KM product limit estimator.

### 3.3 Safety endpoints

In the primary analysis, the safety profile of the combination of voriconazole and anidulafungin was similar to that of voriconazole monotherapy, except for a greater proportion of patients in the combination group had hepatobiliary AEs than in the monotherapy group (12.7% vs 8.4%).^13^

The overall frequencies of TEAEs of all causalities in both populations are shown in Table 4 and by system organ class in Table 5. In this post hoc analysis, the most common TEAEs in the Korean sub‐group were rash (combination therapy [N = 28]: 28.6% and monotherapy [N = 28]: 25.0%), constipation (combination therapy: 25.0% and monotherapy: 21.4%), nausea (combination therapy: 25.0% and monotherapy: 14.3%) and hypokalaemia (combination therapy: 17.9% and monotherapy: 21.4%). In the non‐Korean sub‐group, the most common TEAEs were pyrexia (combination therapy [N = 200]: 15.0% and monotherapy [N = 198]: 20.7%), diarrhoea (combination therapy: 17.5% and monotherapy: 17.7%) and nausea (combination therapy: 15.5% and monotherapy: 19.2%) (Appendix S1: Table S2).

**Table 4:** Treatment‐emergent adverse events of all causalities in Korean and non‐Korean patients (safety population)

	Voriconazole + anidulafungin	Voriconazole + placebo
Korean patients	N = 28	N = 28
Total number of AEs	216	227
Patients with AEs, n (%)	26 (92.9)	27 (96.4)
Patients with SAEs, n (%)	16 (57.1)	13 (46.4)
Patients discontinuing due to AEs, n (%)	2 (7.1)	3 (10.7)
Patients with dose reductions or temporary discontinuations due to AEs, n (%)	6 (21.4)	1 (3.6)
Non‐Korean patients	N = 200	N = 198
Total number of AEs	1743	1650
Patients with AEs, n (%)	193 (96.5)	192 (97.0)
Patients with SAEs, n (%)	99 (49.5)	91 (46.0)
Patients discontinuing due to AEs, n (%)	10 (5.0)	13 (6.6)
Patients with dose reductions or temporary discontinuations due to AEs, n (%)	29 (14.5)	19 (9.6)

Abbreviations: AE, adverse event; n, number of patients; SAE, serious adverse event.

**Table 5:** Treatment‐emergent adverse events by system organ class occurring with a frequency of > 5% in Korean and non‐Korean patients (safety population)

Category	Voriconazole + anidulafungin		Voriconazole + placebo	
	Korean patients, n (%) (N = 28)	Non‐Korean patients, n (%) (N = 200)	Korean patients, n (%) (N = 28)	Non‐Korean patients, n (%) (N = 198)
Blood and lymphatic system disorders	4 (14.3)	32 (16.0)	5 (17.9)	38 (19.2)
Cardiac disorders	4 (14.3)	39 (19.5)	6 (21.4)	39 (19.7)
Ear and labyrinth disorders	0	7 (3.5)	2 (7.1)	5 (2.5)
Eye disorders	10 (35.7)	39 (19.5)	7 (25.0)	51 (25.8)
Gastrointestinal disorders	15 (53.6)	115 (57.5)	18 (64.3)	115 (58.1)
General disorders and administration site conditions	11 (39.3)	109 (54.5)	14 (50.0)	114 (57.6)
Hepatobiliary disorders	4 (14.3)	25 (12.5)	5 (17.9)	14 (7.1)
Infections and infestations	12 (42.9)	93 (46.5)	12 (42.9)	87 (43.9)
Injury, poisoning and procedural complications	4 (14.3)	31 (15.5)	1 (3.6)	21 (10.6)
Investigations	8 (28.6)	70 (35.0)	5 (17.9)	62 (31.3)
Metabolism and nutrition disorders	16 (57.1)	75 (37.5)	12 (42.9)	70 (35.4)
Musculoskeletal and connective tissue disorders	3 (10.7)	47 (23.5)	3 (10.7)	40 (20.2)
Neoplasms benign, malignant and unspecified (including cysts and polyps)	1 (3.6)	24 (12.0)	2 (7.1)	10 (5.1)
Nervous system disorders	5 (17.9)	60 (30.0)	10 (35.7)	53 (26.8)
Psychiatric disorders	9 (32.1)	68 (34.0)	7 (25.0)	66 (33.3)
Renal and urinary disorders	9 (32.1)	39 (19.5)	8 (28.6)	34 (17.2)
Reproductive system and breast disorders	0	3 (1.5)	2 (7.1)	2 (1.0)
Respiratory, thoracic and mediastinal disorders	14 (50.0)	96 (48.0)	12 (42.9)	94 (47.5)
Skin and subcutaneous tissue disorders	15 (53.6)	68 (34.0)	12 (42.9)	67 (33.8)

Note

Data are n (%).

The mortality rate in Korean patients (safety population) at Week 6 was 14.7% (4/28) in the combination therapy arm and 32.1% (9/28) in the monotherapy arm, with a similar pattern at Week 12 (Appendix S1: Table S3). In the non‐Korean population, the mortality rates at Week 6 were similar in the two therapy arms, respectively (21.0% and 22.6%) (Appendix S1: Table S3).

## 4 DISCUSSION

This study was one of the first large randomised, controlled trials to assess combination therapy for the treatment of IA. The results of the primary study showed that combination therapy with anidulafungin and voriconazole was associated with a numerically higher, but not statistically significant, reduction in overall mortality.^13^ In the investigation, a relatively homogeneous population was enrolled, who were considered to have a lower risk for death because of their underlying conditions. The entry criteria were designed to measure treatment in patients with IA who had a low risk for death.^13^

In our post hoc analysis, we have shown that in Korean patients with IA, combination antifungal therapy with anidulafungin and voriconazole was associated with better outcomes at Weeks 6 and 12 than with voriconazole monotherapy. The benefit of combination therapy over monotherapy after 6 weeks appeared to be greater for Korean than non‐Korean patients included in the mITT population. This is an interesting finding, because studies have shown that a higher proportion of the Asian population, including Koreans, have the poor metabolizer CYP2C19 genotype, which can lead to increased trough voriconazole concentration, compared with Caucasians.^18^, ^19^, ^20^, ^21^, ^22^, ^26^ An antagonistic interaction has been observed between anidulafungin and voriconazole at higher exposures, suggesting that reduced efficacy may be observed in poor metabolizers receiving combination therapy compared with poor metabolisers receiving similar doses of monotherapy or non‐poor metabolizers receiving combination therapy.^27^ Koreans in our study had lower weight overall compared with non‐Koreans. Although weight is generally thought to influence response to therapies, a pharmacokinetic‐pharmacodynamic study of voriconazole in patients with IA did not determine weight as a predictor of efficacy.^28^, ^29^

As well as being used in the diagnosis of IA, a serum test for GM can help to predict clinical outcome in patients with probable or proven IA in patients with haematologic malignancies.^16^, ^30^ Multivariate analysis of data in a prospective, open‐label observational study of Korean patients showed that positive GM tests before initiation of empirical antifungal therapy with itraconazole and abnormal findings on chest X‐rays or computed tomography scans were significantly associated with poor outcomes of empirical antifungal therapy with itraconazole.^31^ In our study, all‐cause mortality in Korean patients with baseline GM value > 2.0 at Week 6 was 3/4 and 5/10 in the combination therapy and voriconazole monotherapy groups, respectively.

In the primary voriconazole and anidulafungin combination vs voriconazole monotherapy study, serum or BAL GM‐positive baseline tests were noted in 80% of the combination therapy group and 77.5% of the monotherapy group.^13^ The majority of patients in the Korean sub‐group had serum or BAL GM‐positive baseline tests (71.4% combination therapy; 60.7% monotherapy). Also, for baseline GM values in the range of > 0.5‐2.0, mortality at Week 6 in Korean patients (mITT) was 0/13 after combination therapy and 2/6 after monotherapy. This suggests that the Korean population may have a higher likelihood of IA by positive baseline GM than the overall study population. However, IA was mostly diagnosed by host factor, radiological finding and GM test results in Korea at the time of the study. Fungus culture from BAL or sputum was not routinely carried out in cases of suspected contamination, and other procedures such as biopsy were not performed during the study period, due to bleeding risk. This could account for the apparent relationship between IA risk and baseline GM. Moreover, anidulafungin, which inhibits fungal cell wall synthesis,^32^ is thought to be more effective than voriconazole at lowering GM release to the blood or the lungs, although research comparing triazoles with other antifungal agents is currently limited.^33^ In the 2017 ESCMID‐ECMM‐ERS clinical guideline, combination therapy was not recommended as a primary treatment for aspergillosis; combined voriconazole and anidulafungin had CI (grade/level) evidence for use in patients undergoing allogenic HSCT without neutropenia. However, voriconazole and echinocandin combination therapy had an AIII grade of evidence for use in patients with documented resistance to azoles.^34^ A case of azole‐resistant IA in Korea was recently documented.^35^ Because of wide use of mould‐active azole prophylaxis and its potential threat or azole‐resistant and/or breakthrough IA, combination strategies with voriconazole and echinocandin for the management of IA would be recommended in these situations.^34^ The combination of anidulafungin and voriconazole may be more effective than voriconazole in the Korean than the non‐Korean population, as this Korean sub‐group was more responsive to the GM‐lowering effect of voriconazole and anidulafungin, especially when the baseline GM value was in the range of > 0.5‐2.0. Notably, in our post hoc analysis, in individual Korean patients, GM values showed a general decline over time compared with baseline in the combination therapy and monotherapy arms, regardless of survival outcomes. However, the sample size is too small to draw conclusions.

The safety profiles of the combination therapy and monotherapy in both Korean and non‐Korean populations were compatible, and TEAEs by system organ class mirrored that observed in the overall population.^13^ TEAEs with similar frequencies were observed across both Korean and non‐Korean patients in both treatment groups. The most common TEAEs were rash, constipation, nausea and hypokalaemia in Korean patients and pyrexia, diarrhoea and nausea in non‐Korean patients. Temporary discontinuations or dose reductions were higher in the combination therapy group than in the monotherapy group and were higher in Koreans than non‐Koreans in the combination therapy group. This could be due to a higher proportion of Korean than non‐Korean patients with the poor metabolizer CYP2C19 genotype, resulting in higher trough voriconazole concentrations that may lead to increased AEs.^29^ However, it is difficult to draw meaningful comparisons between the two groups due to the small number of Korean patients in the study. For the safety population, in Korean patients, all‐cause mortality at Weeks 6 and 12 was lower in the combination therapy arm than in the monotherapy arm. In contrast, all‐cause mortality at Week 6 was comparable in the two therapy arms in non‐Korean patients.

The limitations of the primary study have been acknowledged.^13^ The present study was a post hoc analysis and was not pre‐planned in the primary study protocol; it therefore inherently has limitations related to both the post hoc analysis and the lower population. Numerical differences reported in the study may not represent statistical significance as overall numbers in the Korean sub‐group were small, which also precluded multivariate analyses. Additionally, the post hoc data assessment was not blinded, although the original findings were obtained by independent blinded assessment. Finally, therapeutic drug monitoring or analysis of voriconazole and anidulafungin serum levels was not conducted in this study. This would be interesting to explore in future studies, particularly to further our understanding of voriconazole metabolism (in combination or monotherapy) in Korean patients with the poor metabolizer CYP2C19 genotype.

In summary, in Korean patients with IA, a trend was observed in favour of combination therapy over monotherapy. Also, Korean patients benefited slightly more with combination therapy than non‐Korean patients, which may be due to Korean investigators adopting a diagnostic‐driven approach in IA treatment that may be more beneficial for IA treatment from a risk‐benefit perspective. It may also be due to Korean patients being more susceptible to the GM‐lowering effect of voriconazole and anidulafungin when baseline GM values were in the range of > 0.5‐2.0. However, due to the post hoc nature of the analysis, a larger, randomised study in Korean patients is required to confirm these findings.

## CONFLICT OF INTEREST

D‐GL has received grants and personal fees from Astellas, Gilead Sciences, MSD, Pfizer and Yuhan, outside the submitted work. H‐JL is an employee of Pfizer Inc, Seoul. JLY, SS‐FL and JAA are employees of Pfizer Inc, New York.

## AUTHOR CONTRIBUTIONS

D‐GL contributed to the design of the study, analysis and interpretation of the data, drafting and reviewing of the manuscript and final approval. H‐JL contributed to the initiation of the study concept and data interpretation, drafting and reviewing of the manuscript and final approval. JLY contributed to the analysis and interpretation of the data, drafting and reviewing the manuscript and final approval. SS‐FL contributed to the design of the study, analysis and interpretation of the data, drafting and reviewing of the manuscript and final approval. JAA contributed to the design of the study, analysis and interpretation of the data, drafting and reviewing of the manuscript and final approval.

## RESEARCH DATA SHARING

Upon request, and subject to certain criteria, conditions and exceptions (see https://www.pfizer.com/science/clinical-trials/trial-data-and-results for more information), Pfizer will provide access to individual de‐identified participant data from Pfizer‐sponsored global interventional clinical studies conducted for medicines, vaccines and medical devices (a) for indications that have been approved in the US and/or EU or (b) in programmes that have been terminated (ie development for all indications has been discontinued). Pfizer will also consider requests for the protocol, data dictionary and statistical analysis plan. Data may be requested from Pfizer trials 24 months after study completion. The de‐identified participant data will be made available to researchers whose proposals meet the research criteria and other conditions, and for which an exception does not apply, via a secure portal. Data requestors must enter into a data access agreement with Pfizer to gain access.
