# Supplementary material for: Efficacy and safety of combination antifungal therapy in Korean haematological patients with invasive aspergillosis
Source: Mycoses. 2019 Aug 18;62(10):969–78. doi: 10.1111/myc.12972 (PMC7003761; doi:10.1111/myc.12972)
Supplement: Supplementary file 1 [file MYC-62-969-s001.docx]

# Supplementary material

## Supplementary methods

Secondary endpoints in the primary analysis included:

- rate of global response at 6 weeks
- all-cause mortality at 6 weeks in patients with possible, probable or proven invasive aspergillosis (IA) (intent-to-treat [ITT] population: consisting of the modified ITT [mITT] group plus patients with possible IA that could not be upgraded to probable or proven IA within 7 days)
- all-cause mortality at 12 weeks in patients with probable or proven IA
- mortality due to IA at 6 weeks
- time to death due to IA
- time to death (all-cause mortality)
- safety and tolerability of voriconazole monotherapy compared to that of voriconazole in combination with anidulafungin.

**Table S1** European Organisation for Research and Treatment of Cancer/Mycoses Study Group (EORTC/MSG) consensus definitions^a^

| **Proven IA** | **Probable IA** | **Possible IA** |
| --- | --- | --- |
| Histopathologic, cytopathologic or direct microscopic examination of a needle aspiration or biopsy specimen showing hyphal forms with evidence of associated tissue damage (either microscopically or as an infiltrate or lesion by imaging)  **OR**  recovery of *Aspergillus* species by culture from a sample obtained by a sterile procedure from a normally sterile and clinically or radiographically abnormal site consistent with an infectious disease process, excluding BAL, cranial sinus cavity and urine | **One host factor** i.e. recent history of neutropenia, allogeneic HSCT, treatment with T‑cell immune suppressants, prolonged corticosteroid use or inherited severe immune-deficiency  **AND**  **one clinical criterion** i.e. evidence of lower respiratory, tracheobronchial, sinonasal, ophthalmologic or CNS disease  **AND**  **one microbiological criterion** i.e. cytology, direct microscopy, culture OR two serum samples/one BAL sample positive for galactomannan (cut-off of 0.5) | **One host factor**  **AND**  **one clinical criterion**  **NOTE:** Patients enrolled with possible IA **were required to have had a proven or probable diagnosis established within 7 days of enrolment** |

^a^de Pauw B, Walsh TJ, Donnelly JP, et al. Revised definitions of invasive fungal disease from the European Organization for Research and Treatment of Cancer/Invasive Fungal Infections Cooperative Group and the National Institute of Allergy and Infectious Diseases Mycoses Study Group (EORTC/MSG) Consensus Group. *Clin Infect Dis.* 2008;46(12):1813-1821.
BAL, bronchoalveolar lavage; CNS, central nervous system; HSCT, haematopoietic stem cell transplantation; IA, invasive aspergillosis.

**Table S2** Treatment-emergent adverse events by system organ class and MedDRA-preferred terms occurring with a frequency of >5% in Korean and non-Korean patients (safety population)

| Category | Voriconazole + anidulafungin | | Voriconazole + placebo | |
| --- | --- | --- | --- | --- |
|  | **Korean patients (N = 28)** | **Non-Korean patients (N = 200)** | **Korean patients (N = 28)** | **Non-Korean patients (N = 198)** |
| Blood and lymphatic system disorders | 4 (14.3) | 32 (16.0) | 5 (17.9) | 38 (19.2) |
| Anaemia | 0 | 9 (4.5) | 1 (3.6) | 12 (6.1) |
| Febrile neutropenia | 0 | 6 (3.0) | 2 (7.1) | 12 (6.1) |
| Neutropenia | 3 (10.7) | 7 (3.5) | 2 (7.1) | 5 (2.5) |
| Cardiac disorders | 4 (14.3) | 39 (19.5) | 6 (21.4) | 39 (19.7) |
| Atrial fibrillation | 1 (3.6) | 8 (4.0) | 2 (7.1) | 6 (3.0) |
| Pericardial effusion | 2 (7.1) | 1 (0.5) | 0 | 2 (1.0) |
| Tachycardia | 0 | 14 (7.0) | 2 (7.1) | 19 (9.6) |
| Ear and labyrinth disorders | 0 | 7 (3.5) | 2 (7.1) | 5 (2.5) |
| Eye disorders | 10 (35.7) | 39 (19.5) | 7 (25.0) | 51 (25.8) |
| Dry eye | 3 (10.7) | 4 (2.0) | 0 | 5 (2.5) |
| Vision blurred | 4 (14.3) | 10 (5.0) | 0 | 11 (5.6) |
| Visual impairment | 2 (7.1) | 7 (3.5) | 4 (14.3) | 7 (3.5) |
| Gastrointestinal disorders | 15 (53.6) | 115 (57.5) | 18 (64.3) | 115 (58.1) |
| Abdominal distension | 0 | 4 (2.0) | 2 (7.1) | 5 (2.5) |
| Abdominal pain | 0 | 14 (7.0) | 2 (7.1) | 11 (5.6) |
| Abdominal pain upper | 0 | 9 (4.5) | 1 (3.6) | 14 (7.1) |
| Constipation | 7 (25.0) | 28 (14.0) | 6 (21.4) | 24 (12.1) |
| Diarrhoea | 4 (14.3) | 35 (17.5) | 5 (17.9) | 35 (17.7) |
| Dyspepsia | 2 (7.1) | 4 (2.0) | 6 (21.4) | 6 (3.0) |
| Gingival swelling | 0 | NR | 2 (7.1) | NR |
| Haemorrhoids | 0 | 2 (1.0) | 2 (7.1) | 2 (1.0) |
| Nausea | 7 (25.0) | 31 (15.5) | 4 (14.3) | 38 (19.2) |
| Oral pain | 0 | 0 | 2 (7.1) | 1 (0.5) |
| Stomatitis | 1 (3.6) | 3 (1.5) | 2 (7.1) | 4 (2.0) |
| Vomiting | 2 (7.1) | 31 (15.5) | 3 (10.7) | 21 (10.6) |
| General disorders and administration site conditions | 11 (39.3) | 109 (54.5) | 14 (50.0) | 114 (57.6) |
| Asthenia | NR | 9 (4.5) | NR | 10 (5.1) |
| Catheter site pain | 2 (7.1) | 0 | 1 (3.6) | 4 (2.0) |
| Chest pain | 1 (3.6) | 16 (8.0) | 0 | 9 (4.6) |
| Chills | 1 (3.6) | 12 (6.0) | 3 (10.7) | 7 (3.5) |
| Face oedema | 1 (3.6) | 3 (1.5) | 2 (7.1) | 3 (1.5) |
| Fatigue | NR | 6 (3.0) | NR | 14 (7.1) |
| Generalized oedema | 2 (7.1) | 4 (2.0) | 1 (3.6) | 9 (4.6) |
| Mucosal inflammation | 2 (7.1) | 8 (4.0) | 0 | 15 (7.6) |
| Oedema | 1 (3.6) | 11 (5.5) | 4 (14.3) | 10 (5.1) |
| Oedema peripheral | 1 (3.6) | 33 (16.5) | 4 (14.3) | 30 (15.2) |
| Pain | 1 (3.6) | 9 (4.5) | 4 (14.3) | 9 (4.6) |
| Pyrexia | 1 (3.6) | 30 (15.0) | 5 (17.9) | 41 (20.7) |
| Hepatobiliary disorders | 4 (14.3) | 25 (12.5) | 5 (17.9) | 14 (7.1) |
| Hepatitis toxic | 2 (7.1) | NR | 0 | NR |
| Hyperbilirubinaemia | 1 (3.6) | 8 (4.0) | 2 (7.1) | 2 (1.0) |
| Infections and infestations | 12 (42.9) | 93 (46.5) | 12 (42.9) | 87 (43.9) |
| Aspergillosis | 2 (7.1) | 5 (2.5) | 1 (3.6) | 4 (2.0) |
| Bacteraemia | 2 (7.1) | 7 (3.5) | 0 | 10 (5.1) |
| Pneumonia | 2 (7.1) | 8 (4.0) | 2 (7.1) | 10 (5.1) |
| Sepsis | 1 (3.6) | 13 (6.5) | 2 (7.1) | 10 (5.1) |
| Septic shock | 5 (17.9) | 6 (3.0) | 4 (14.3) | 12 (6.1) |
| Sinusitis | 1 (3.6) | 2 (1.0) | 0 | 2 (1.0) |
| Injury, poisoning and procedural complications | 4 (14.3) | 31 (15.5) | 1 (3.6) | 21 (10.6) |
| Excoriation | 2 (7.1) | 1 (0.5) | 0 | 2 (1.0) |
| Fall | NR | 13 (6.5) | NR | 4 (2.0) |
| Procedural pain | 2 (7.1) | 3 (1.5) | 0 | 2 (1.0) |
| Investigations | 8 (28.6) | 70 (35.0) | 5 (17.9) | 62 (31.3) |
| Alanine aminotransferase increased | 1 (3.6) | 10 (5.0) | 0 | 8 (4.0) |
| Aspartate aminotransferase increased | 1 (3.6) | 11 (5.5) | 0 | 13 (6.6) |
| Blood alkaline phosphatase increased | NR | 20 (10.0) | NR | 6 (3.0) |
| Cytomegalovirus test positive | 2 (7.1) | 1 (0.5) | 2 (7.1) | 0 |
| Gamma-glutamyltransferase increased | NR | 11 (5.5) | NR | 10 (5.1) |
| Hepatic enzyme increased | 2 (7.1) | 6 (3.0) | 0 | 7 (3.5) |
| Liver function test abnormal | 3 (10.7) | 7 (3.5) | 0 | 5 (2.5) |
| Metabolism and nutrition disorders | 16 (57.1) | 75 (37.5) | 12 (42.9) | 70 (35.4) |
| Decreased appetite | 4 (14.3) | 11 (5.5) | 1 (3.6) | 13 (6.6) |
| Hypernatraemia | 1 (3.6) | 4 (2.0) | 2 (7.1) | 1 (0.5) |
| Hypoalbuminaemia | 2 (7.1) | 7 (3.5) | 2 (7.1) | 3 (1.5) |
| Hypocalcaemia | 2 (7.1) | 9 (4.5) | 0 | 6 (3.0) |
| Hypoglycaemia | 0 | 7 (3.5) | 2 (7.1) | 2 (1.0) |
| Hypokalaemia | 5 (17.9) | 32 (16.0) | 6 (21.4) | 25 (12.6) |
| Hypomagnesaemia | 4 (14.3) | 14 (7.0) | 1 (3.6) | 14 (7.1) |
| Hypophosphataemia | 2 (7.1) | 8 (4.0) | 1 (3.6) | 10 (5.1) |
| Musculoskeletal and connective  tissue disorders | 3 (10.7) | 47 (23.5) | 3 (10.7) | 40 (20.2) |
| Arthralgia | 2 (7.1) | 8 (4.0) | 0 | 11 (5.6) |
| Back pain | 0 | 13 (6.5) | 2 (7.1) | 10 (5.1) |
| Pain in extremity | 0 | 16 (8.0) | 1 (3.6) | 10 (5.1) |
| Neoplasms benign, malignant and unspecified (including cysts and polyps) | 1 (3.6) | 24 (12.0) | 2 (7.1) | 10 (5.1) |
| Nervous system disorders | 5 (17.9) | 60 (30.0) | 10 (35.7) | 53 (26.8) |
| Dizziness | 1 (3.6) | 6 (3.0) | 2 (7.1) | 9 (4.6) |
| Headache | 2 (7.1) | 14 (7.0) | 3 (10.7) | 23 (11.6) |
| Somnolence | 0 | 9 (4.5) | 4 (14.3) | 3 (1.5) |
| Tremor | NR | 10 (5.0) | NR | 4 (2.0) |
| Psychiatric disorders | 9 (32.1) | 68 (34.0) | 7 (25.0) | 66 (33.3) |
| Agitation | NR | 13 (6.5) | NR | 7 (3.5) |
| Anxiety | 3 (10.7) | 9 (4.5) | 0 | 15 (7.6) |
| Confusional state | 0 | 10 (5.0) | 1 (3.6) | 15 (7.6) |
| Depression | 3 (10.7) | 2 (1.0) | 0 | 6 (3.0) |
| Hallucination | NR | 10 (5.0) | NR | 9 (4.6) |
| Hallucination, visual | 0 | 8 (4.0) | 2 (7.1) | 10 (5.1) |
| Insomnia | 4 (14.3) | 25 (12.5) | 4 (14.3) | 18 (9.1) |
| Renal and urinary disorders | 9 (32.1) | 39 (19.5) | 8 (28.6) | 34 (17.2) |
| Cystitis haemorrhagic | 0 | 2 (1.0) | 2 (7.1) | 0 |
| Dysuria | 1 (3.6) | 3 (1.5) | 3 (10.7) | 4 (2.0) |
| Haematuria | 3 (10.7) | 6 (3.0) | 1 (3.6) | 7 (3.5) |
| Urinary retention | 1 (3.6) | 4 (2.0) | 2 (7.1) | 4 (2.0) |
| Reproductive system and breast disorders | 0 | 3 (1.5) | 2 (7.1) | 2 (1.0) |
| Respiratory, thoracic and mediastinal disorders | 14 (50.0) | 96 (48.0) | 12 (42.9) | 94 (47.5) |
| Cough | 1 (3.6) | 15 (7.5) | 3 (10.7) | 19 (9.6) |
| Dyspnoea | 3 (10.7) | 12 (6.0) | 3 (10.7) | 25 (12.6) |
| Epistaxis | 2 (7.1) | 18 (9.0) | 2 (7.1) | 18 (9.1) |
| Haemoptysis | 2 (7.1) | 10 (5.0) | 3 (10.7) | 6 (3.0) |
| Hiccups | 2 (7.1) | 5 (2.5) | 0 | 3 (1.5) |
| Pleural effusion | 2 (7.1) | 9 (4.5) | 0 | 11 (5.6) |
| Productive cough | 2 (7.1) | 7 (3.5) | 0 | 4 (2.0) |
| Respiratory failure | 0 | 13 (6.5) | 1 (3.6) | 14 (7.1) |
| Rhinorrhoea | 3 (10.7) | 5 (2.5) | 1 (3.6) | 2 (1.0) |
| Skin and subcutaneous tissue disorders | 15 (53.6) | 68 (34.0) | 12 (42.9) | 67 (33.8) |
| Decubitus ulcer | 2 (7.1) | 7 (3.5) | 0 | 4 (2.0) |
| Petechiae | 0 | 5 (2.5) | 1 (3.6) | 11 (5.6) |
| Pruritus | 3 (10.7) | 7 (3.5) | 5 (17.9) | 4 (2.0) |
| Rash | 8 (28.6) | 22 (11.0) | 7 (25.0) | 24 (12.1) |
| Vascular disorders | 7 (25.0) | 69 (34.5) | 6 (21.4) | 48 (24.2) |
| Hypertension | 3 (10.7) | 29 (14.5) | 1 (3.6) | 20 (10.1) |
| Hypotension | 4 (14.3) | 28 (14.0) | 4 (14.3) | 16 (8.1) |

MedDRA, Medical Dictionary for Regulatory Activities; NR, not recorded.

Data are n (%)

**Table S3** All-cause mortality at Week 6 in Korean and non-Korean patients and at Week 12 in Korean patients (safety population)

|  | **Voriconazole + anidulafungin** | **Voriconazole + placebo** |
| --- | --- | --- |
| Sub-group, N | 225 | 226 |
| Week 6 |  |  |
| Korean patients, n^a^ Number of deaths Mortality rate, % | 28 4 14.7 | 28 9 32.1 |
| Non-Korean patients, n^a^ Number of deaths Mortality rate,^b^ % | 197 41 21.0 | 198 44 22.6 |
| Week 12 |  |  |
| Korean patients, n Number of deaths Mortality rate, % | 28 6 22.5 | 28 10 35.7 |

^a^Treatment difference is based on a weighted difference in proportion.
^b^Mortality rate is based on the Kaplan-Meier product limit estimator.


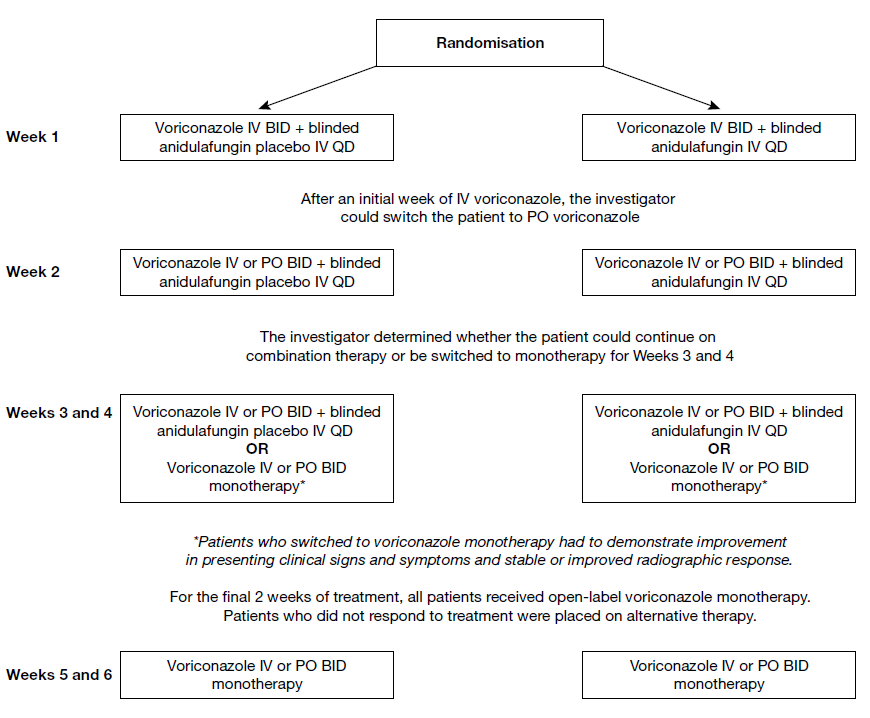
**FIGURE S1.** Primary study overview^a^

^a^Marr KA, Schlamm HT, Herbrecht R, et al. Combination antifungal therapy for invasive aspergillosis: a randomized trial. *Ann Intern Med*. 2015;162(2):81-89.
BID, twice daily; IV, intravenous; PO, orally; QD, every day.
